# PATIENT PRIORITIES CARE: GOAL-ALIGNED CARE FOR VETERANS WITH DEMENTIA AND THEIR CAREGIVERS

**DOI:** 10.1093/geroni/igz038.445

**Published:** 2019-11-08

**Authors:** Jennifer Freytag, Lilian Dindo, Angela Catic, Aanand Naik

**Affiliations:** 1 Center for Innovations in Quality, Effectiveness and Safety, Houston, Texas, United States; 2 Michael E DeBakey VA Medical Center, Houston, Texas, United States

## Abstract

Patient Priorities Care (PPC) is an approach to clinical conversations that focuses on aligning care with patient priorities. This approach involves identifying and incorporating patient values, goals, and preferences into care for patients with multiple comorbid conditions. We have previously described how this clinically feasible and sustainable approach has been implemented in VA and private healthcare settings, and how this approach results in documented care aligned with patient goals. In this paper, we describe the way PPC has been adapted and implemented in a VA geriatrics clinic for Veterans with dementia and their caregivers. We discuss results from a funded pilot project in which we investigated the role of caregivers in patient goal setting; assessed patient functioning using self-reported and sensor measures; and examined clinical conversations and interviews. We use these results to discuss implementation strategies targeting: (1) caregiver activation and involvement in clinical conversation and decision-making, (2) patient and caregiver education about the disease trajectory of dementia, (3) life space function and using physical sensor data in dementia care, (4) measures of community integration for patients and caregivers, and (5) features of successful clinical conversations in this context.

